# Neurodevelopment and Endocrine Disruption

**DOI:** 10.1289/ehp.6601

**Published:** 2003-11-17

**Authors:** Theo Colborn

**Affiliations:** Department of Zoology, University of Florida, Gainesville, Florida, USA, and The Endocrine Disruption Exchange, Paonia, Colorado, USA

**Keywords:** ADHD, autism, behavior, endocrine disruptor, environmental contaminants, neurologic effects, prenatal exposure, thyroid

## Abstract

In this article I explore the possibility that contaminants contribute to the increasing prevalence of attention deficit hyperactivity disorder, autism, and associated neurodevelopmental and behavioral problems in developed countries. I discuss the exquisite sensitivity of the embryo and fetus to thyroid disturbance and provide evidence of human *in utero* exposure to contaminants that can interfere with the thyroid. Because it may never be possible to link prenatal exposure to a specific chemical with neurodevelopmental damage in humans, I also present alternate models where associations have been made between exposure to specific chemicals or chemical classes and developmental difficulties in laboratory animals, wildlife, and humans.

Approximately 12 years ago the scientific community acknowledged that certain synthetic chemicals are capable of crossing the placental and brain barriers and interfering with development and function (Colborn and Clement 1992). The chemicals mimic or interfere with endogenous hormones and other signaling chemicals of the endocrine system. These chemicals, distinguished as endocrine disruptors, bridge many chemical classes and are an integral part of the world economy and commerce. To date no validated or standardized screens or assays have been developed to test chemicals for their possible endocrine-disrupting effects. Consequently, none of the thousands of chemicals used today have been tested systematically for these effects for regulatory purposes. Despite this, the list is growing of known endocrine disruptors having a wide range of mechanisms of action that can interfere with brain development (Brucker-Davis 1998; Howdeshell 2002).

The production and use of industrial and agricultural chemicals have increased at an almost exponential rate for the past 50 years, with roughly 10 new chemicals currently being introduced each day. The U.S. Environmental Protection Agency (U.S. EPA) estimates that 87,000 chemicals are in use today. In the United States the plastics industry has grown at the rate of 6–12% per year since the mid-1940s, with annual production in the United States reaching 85 billion pounds (> 338 pounds per person per year) in 1996. In developing countries, plastics production is expanding at the rate of 40% per year (Society of the Plastics Industry 1997). Plastics are used in toys, cosmetics, perfumes, cleaning compounds, clothing, telecommunication equipment, computers, almost all household products, high-impact sporting equipment, and construction material from buildings to automobiles, airplanes, and aerospace vehicles.

Currently approximately 875 active ingredients registered as pesticides by the U.S. EPA have been formulated into 21,000 pesticide products, with many more new products entering the market each month (Short and Colborn 1999). In 1995 the United States produced 1.3 billion pounds of pesticide active ingredients, of which herbicides (weed killers) are the most widely used. It is estimated that herbicides cover > 14% of the land surface of the United States. This does not include nonfarm use for lawns, gardens, golf courses, parks, roadsides, railways, airports, forests, federal applications on government lands, and vast rights-of-way by states, counties, and municipalities. More than 60% of herbicides are documented endocrine disruptors (Short and Colborn 1999). Among the most widely used herbicides that interfere with the thyroid system are 2,4-dichlorophenoxyacetic acid (2,4-D), acetochlor, aminotriazole, amitrole, bromacil, bromoxynil, pendamethalin, and the thioureas (Brucker-Davis 1998; Howdeshell 2002; Short and Colborn 1999).

## Historical Perspective of Exposure and Human Disorders

When data on the growth in synthetic chemical production are compared with the data on increasing prevalence of neurodevelopmental and other developmental disorders in humans, the data begin to merge around 1970. At approximately the same time, the first generation of humans exposed in the womb to synthetic chemicals on a large scale began to have children of their own (Table 1). For example, a plastic monomer, bisphenol A (BPA), was introduced in the early 1920s. Polychlorinated biphenyls (PCBs) were introduced in 1929. DDT became available for retail sale in 1938, and the large-scale, widespread commercial use of a vast number of synthetic chemicals commenced near the end of World War II (WWII) in the 1940s. Companies previously producing chemicals for warfare converted to making pesticides and plastics as the petroleum industry began to find more uses for its by-products from gasoline production. Although indivduals were being exposed to these chemicals since the early 1920s, it was not until the end of WWII that exposure increased to such an extent that vast numbers of adults exposed daily were accumulating significant amounts of these chemicals in their bodies. In terms of generation time, these individuals in the 1950s produced the first generation of offspring exposed to numerous synthetic chemicals in the womb and at increased levels. By 1970 these post-WWII babies were having children of their own. It was during the 1970s that what appeared to be increases in unusual, previously rare neurodevelopmental disorders began to catch the attention of health professionals.

Terms such as learning disabilities, autism, attention deficit hyperactivity disorder (ADHD), childhood cancers, juvenile diabetes, and juvenile delinquency became household words by the mid-1990s. Parental support groups emerged across the nation for each anomaly, and in response, health authorities began to acknowledge these increases. In 1995 the U.S. EPA established the Children’s Environmental Health Program to develop preventive measures to protect children from exposure to environmental contaminants, and in 2000, a presidential initiative led to the establishment of children’s health centers nationwide to develop treatments and cures for these problems.

Gershon and Rieder (1992) provided one of the earliest alerts that neurodevelopmental problems were increasing in the United States. They reported that suicides were 10-fold higher in teenagers 15–19 years of age born in the 1950s than those born in the 1930s (Gershon and Rieder 1992). In addition the rates of depression and mania were continuing to rise with each new birth cohort they examined. They called it a “mystery.” They wrote that mood disorders could arise from the interactions between genes and “some aspects of the environment.” They implied that some individuals were more susceptible to environmental stresses than others because of their genetic makeup. Although it was suggested that the increases reflected better diagnoses, there was still much discussion in the media and among parents. Then, in 1987, Berkow and Fletcher (1987) estimated that as many as 10% of the children < 13 years of age in the United States suffered ADHD. A study by Weiss et al. (1993) on children born between 1979 and 1982 found thyroid abnormalities 5 times more frequent in children with ADHD. A study by Rowland et al. (2002) on children born in the late 1980s and early 1990s found that 15% of the boys and 5% of the girls from grades 1–5 in a North Carolina countywide school district had been diagnosed with ADHD (*p* < 0.001). The study also found that 11% of the boys and 3% of the girls were on medication. The authors suggested this is an underreported anomaly because sampling depended solely on parental responses.

In the 1980s, occasional reports concerning the increased prevalence of autism began to appear in the peer-reviewed literature from the United States and other countries. More accurate clinical diagnoses and reporting of autism occurred after the American Psychiatric Association defined this syndrome in 1994 (American Psychiatric Association 1994). Although estimates on the prevalence of the disorder vary widely depending on the scope of the definition of the term, the most recent studies consistently reveal higher prevalence or incidence rates (depending how the study was designed) of both the narrow and broad definitions. Autism disorder includes a limited number of classic symptoms, using a narrow set of criteria, whereas autistic spectrum disorder includes a broader scope of symptoms that has been made possible in recent years with better diagnostic tests and technologies. A 2001 study in the United Kingdom reported 16.8 autistic children per 10,000 using the narrow definition and 62.6 per 10,000 using the broader definition (Chakrabarti and Fombonne 2001). A study in 1998 of Brick Township, New Jersey, found 40 cases per 10,000 in the narrow definition group and 67 per 10,000 in the broader definition group (Bertrand et al. 2001). This study was initiated because of community concern about exposure to industrial emissions. A 2003 study by the U.S. Centers for Disease Control and Prevention (CDC) (Yeargin-Allsopp et al. 2003) found 19–47 cases per 10,000 from a random sampling of children in 1996 between 3 and 10 years of age from five counties in metropolitan Atlanta, Georgia; the male–female ratio was 4:1. As with ADHD, boys are significantly more likely to develop autism than girls. “Autism is the fastest-growing developmental disability, increasing at a rate of 10 to 17 percent annually,” according to the Autism Society of America (Grossman 2002).

By 1996, on the basis of extensive magnetic resonance imaging (MRI) and histological examination in both humans and laboratory animals, it became apparent that the original autism lesion occurs just before or shortly after neural tube closure. This is around week 6 or 7 in the human fetus (Bayer et al. 1993; Howdeshell 2002; Rodier et al. 1996). During this stage of development the large cerebellar neurons begin to develop. The increased brain weight of autistic children, the packing of cells in the limbic system, the deficiency in Purkinje cells and granule cells of the cerebellum, and a number of other differences in the autistic brain suggest that numerous tissues and stages of brain development can be affected as the autistic syndrome evolves. In addition one retrospective study showed that when thalidomide exposure occurred between gestation days 20 and 24 (week 3), approximately 30% of the phocomelia cases were also autistic (Miller and Strömland 1993).

## Sensitivity of Neurodevelopment to Thyroid Hormones

**Figure 1:**
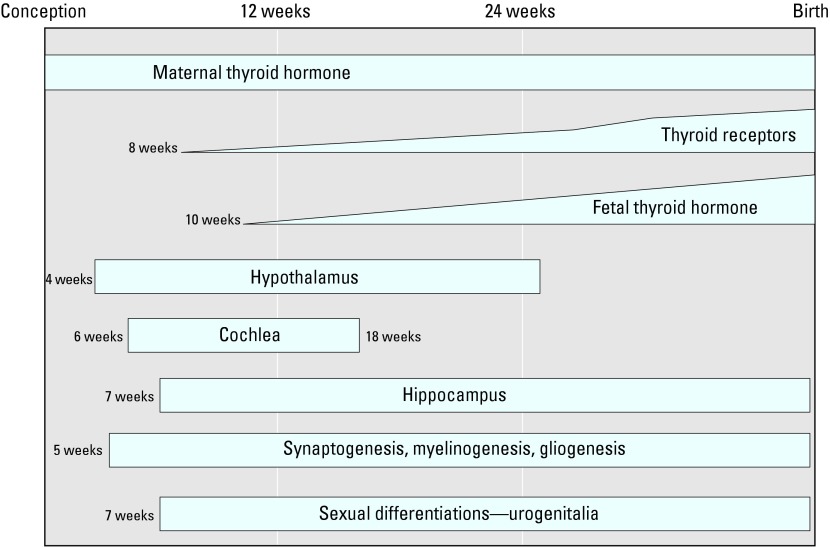
Role of thyroid hormones in fetal neurologic development in relation to timing of several landmark stages of development. Figure adapted from Howdeshell (2002).

Although it has been known for a century that hypothyroidism leads to retardation and other serious developmental effects, the role of thyroid hormones in brain development is still not completely understood (Rice and Barone 2000). It is also accepted that thyroid hormones transferred from the mother to the embryo and fetus are critical for normal brain development (Lazarus 1999), even though the thyroid gland of a fetus starts producing thyroid hormones at about 10 weeks (Shepard 1967) (Figure 1).

We now recognize that only a slight difference in the concentration of thyroid hormones during pregnancy can lead to significant changes in intelligence in children. In pregnant women, normal thyroid hormones circulate bound to protein at parts per billion (ppb) and as free hormone at parts per trillion (ppt). In a long-term study Haddow et al. (1999) collected and stored blood from women in their second trimester of pregnancy. Years later, their children were tested between 7 and 9 years of age for intelligence, attention, language, reading ability, school performance, and visual–motor performance. The data of Haddow et al. revealed a possible association between a relatively slight reduction in the amount of circulating free thyroid hormone levels in the mothers (2.6 ppt) and the intellectual development of their children (Haddow et al. 1999). Children of mothers with a geometric mean of 9.1 ppt free thyroxine (fT_4_) during gestation scored 4 points higher in IQ than children of mothers with a geometric mean of 7.5 ppt (*p* = 0.002). In this study the thyroxine (T_4_) level in the lower IQ cohort was at the low end of what is considered the normal T_4_ range (Haddow et al. 1999). Haddow et al. (1999) were not attempting to link exposure to synthetic chemicals with loss of intelligence in the children. However, their results demonstrate that synthetic chemicals, which can interfere with the thyroid system, would not have to be present in very high concentrations to affect the intellectual and behavioral development of embryos and fetuses. Their study unexpectedly demonstrates the fragile relationship between a mother and her developing offspring.

## Mechanisms of Action of Thyroid-Disrupting Chemicals

For investigators to understand the role of endocrine disruption in brain development, it was necessary to first understand how thyroid hormones regulate brain development. The complexity of the development of both the neurologic and thyroid systems offers numerous opportunities for chemicals to interfere as the systems develop, mature, and function. Howdeshell (2002) provides a current road map about the simultaneous development of the neurologic and thyroid systems (Figure 1). She also provides a list of those synthetic environmental chemicals (aside from pharmaceuticals and designer chemicals) known to interfere with these systems for which mechanism of action has been determined [see Howdeshell (2002) for further list and discussion]. Below is a list of demonstrated thyroid–pituitary disruptions that result from environmental exposure.

- Inhibition of active transport of inorganic iodide into the follicular cell
- Interference with the sodium/iodide transporter system
- Inhibition of thyroid peroxidases to convert inorganic iodide into organic iodide to couple iodinated tyrosyl moieties into thyroid hormone
- Damage to follicular cells
- Inhibition or enhancement of thyroid hormone release into the blood
- Inhibition or activation of the conversion of T_4_ to T_3_ by 5′-monodeiodinase at various sites in the body, for example, the fetal brain
- Enhancement or interference of the metabolism and excretion of thyroid hormone by liver uridine diphosphate
- Interference with transport of thyroid hormones
- Vitamin A (retinol) disturbances
- Blocking of or interfering with thyroid receptors

Briefly, there are chemicals that interfere with iodine uptake (the herbicides 2,4-D and man-cozeb, several PCB congeners, and thiocyanates) and peroxidation at the molecular level (the herbicides aminotriazole and thioureas, the insecticides endosulfan and malathion, and PCBs). They also interfere with the protein transporter that provides a pathway for iodine to enter the cell (military and aerospace chemicals, perchlorates). Certain antagonists (PCBs, the herbicides aminotriazole and dimethoate, and the insecticide fenvalerate) prevent the release of thyroid hormone from the cell and inhibit conversion of T_4_ to triiodothyronine (T_3_). Various chemicals enhance excessive excretion of thyroid hormones, some through activation of the cytochrome P450 system (dioxin, hexa-chlorobenzene, and fenvalerate). Some PCBs, phthalates, and other widely used chemicals compete for sites on the thyroid transport proteins that deliver thyroid hormones throughout the body. New research focuses on the role of chemicals as they interfere with vitamin A (retinols), the retinol receptors, and the essential dimerization of thyroid hormone with retinols, a process essential for thyroid hormone expression. There is still no evidence that environmental chemicals directly block the thyroid receptor.

For years it was thought that in humans transthyretin (TTR) played a special role among the thyroid transport proteins, albumin and thyroglobulin, to transport fT_4_ into the fetal brain where it is converted by the enzyme deiodinase to free T_3_ (fT_3_) (Porterfield 1994). However, recent research using TTR knockout mice reveals that TTR is not necessary for transport of fT_4_ to the fetal murine brain (Palha et al. 2002). Nonetheless, Brouwer et al. (1998) pointed out that during normal enzyme detoxification of PCBs in the maternal liver, certain PCB congeners are hydroxylated. This metabolic step enhances the binding affinity of the hydroxylated PCBs to TTR (Brouwer et al. 1998). Traveling on TTR in the blood, the hydroxylated PCBs cross the placenta, enter the fetus, and ultimately the fetal brain. Through their high-affinity binding the hydroxylated congeners displace essential fT_4_ that must get to the fetal brain to be converted to fT_3_. Schuur et al. (1998) demonstrated that the hydroxylated PCBs also interfere with the normal excretion of thyroid hormones by inhibiting their sulfation. PCB hydroxylates also have estrogenic and antithyroid activity (Iwasaki et al. 2002). For example, developing brain cells exposed to the PCB hydroxylate 4(OH)-2′,3,3′,4′5′-pentachlorobiphenyl (10^−10^ M) displayed the strongest suppression of thyroid hormone–activated transcription compared with any other developing cells tested in an *in vitro* assay (Iwasaki et al. 2002). This is another example of the ultrasensitivity of brain development to PCBs.

At the organism level, U.S. EPA scientists were able to demonstrate the critical role of thyroid hormones in the development of the ear, normal hearing, and motor control. As shown in Figure 1, the cochlea in the human ear begins to form around 6 weeks *in utero*. The cochlea is connected directly with the brainstem, allowing for immediate transmission to and interpretation of sound in the brain. A properly constructed cochlea is critical for hearing. Pregnant rats fed the antithyroid pharmaceutical propyl thiouracil had both difficulty hearing low and intermediate frequency clicking sounds and loss of motor coordination (Goldey et al. 1995b). Another set of pregnant rats was fed Aroclor 1254, a commercial mixture of PCBs, which also induced hypothyroidism and the accompanying hearing and motor problems (Goldey et al. 1995a). To confirm that the PCBs interfered with the thyroid system, pregnant rats were fed Aroclor 1254 supplemented with T_4_, and motor problems in the pups were attenuated (Goldey and Crofton 1998). In line with the U.S. EPA findings, Lavado-Autric et al. (2003) report abnormal cell migration and cytoarchitecture in the hippocampus and primary somatosensory cortex in rat pups whose dams were fed a low-iodine diet. They point out that the pups exhibited detectable functional audio deficits related to this abnormal development (Lavado-Autric et al. 2003).

## Overcoming the Difficulty of Making Causal Links

It is almost impossible to make causal links between prenatal contaminant exposure and developmental damage in humans. Because of this, scientists have used laboratory and wild-animal models to better understand the effects of synthetic chemicals on development. Making a strong association between a particular chemical or class of chemicals and an adverse condition in the field is sometimes difficult, yet in some instances an association can be made by supplementing field research with well-designed laboratory research. For example, reports of serious developmental and reproductive problems among birds in the North American Great Lakes and other regions in developed countries date back to the 1960s and early 1970s (Gilbertson 1975; Leatherland 1992, 1999; Moccia et al. 1986). Thyroid gland and hormone abnormalities in particular were repeatedly reported in Great Lakes herring gulls used in a Canadian monitoring program to track PCBs and other organochlorine chemicals in the lakes. In addition, Leatherland (1992) reported that every top predator fish they examined in the Great Lakes had enlarged thyroid glands. In the late 1990s the problem persisted as the thyroid glands in Lake Erie fish began to rupture because the glands were becoming so large (Leatherland 1999), although the concentrations of a number of the organochlorine chemicals, including the PCBs, had declined considerably in the late 1970s in the Great Lakes. To date, no clear link has been established with a specific chemical in the Great Lakes that causes these thyroid problems. Lack of iodine has been ruled out as a possible link.

Conversely, field observations of damaged brains and spinal cords in bald eagles and great blue herons (Henshel et al. 1993, 1996) led to laboratory experimentation that provided a strong association between the anomalies and 2,3,7,8-tetrachlorodibenzo-*p*-dioxin, which was discovered in elevated levels in the birds. The laboratory results not only provided a causal link with a contaminant but also demonstrated the extreme sensitivity of the developing brain to chemical interference. Myelination is critical for proper nervous system development, and in humans it commences at approximately 12 weeks *in utero* (Figure 1). Myelination was reduced in the spinal cords of chicks exposed to dioxin (10, 100, and 1,000 ppt) in a dose–response manner when injected during their egg stage. Effects were visible and similar to the damage seen in the wild birds at an exposure of 10 ppt, which is within the range of human exposure (Henshel 1998; U.S. EPA 2000).

## The Human Connection

Concern in the 1970s over the widespread health problems among Great Lakes wildlife led to a human epidemiologic study that examined the health effects in infants whose mothers ate two to three meals a month of Lake Michigan fish for at least 6 or more years before their pregnancies (Jacobson and Jacobson 1996). Only healthy mothers and infants were selected for this study. Within 24 hr of birth, significant delays in neuromuscular and neurologic development were detected in the children whose mothers ate the most fish contaminated with PCBs. At 4 years of age some children showed an association between short-term memory problems and the amount of PCBs in the mothers’ blood at delivery. The same children at 11 years of age displayed significantly reduced academic skills accompanied by a mean 6.2-point IQ reduction. This again was associated with their prenatal exposure to PCBs, not the concentration of PCBs in their blood at the time of testing. Although there is no way to prove that PCBs interfered with the development of the cochlea in these children, the affected children had difficulty with audiovisual discrimination and information processing. Some children were as much as 2 years behind their peers in school, were hyperactive, and had attention problems (Jacobson and Jacobson 1996).

Another healthy mother–infant study commenced 12 years later in Oswego, New York, on Lake Ontario to replicate and expand the design of the Lake Michigan study (Darvill et al. 2000). As in the Lake Michigan study, the high fish eaters consumed about the same amount of Lake Ontario fish before their pregnancies (Stewart et al. 1999). Again, prenatal exposure to PCBs was associated with neurodevelopmental changes in their children at approximately 4 years of age. Using another battery of tests, Stewart et al. (2000b) found that the temperaments of the affected children were altered compared with those of lesser-exposed children. They smiled less, were more fearful, and had difficulty adapting to changes in their environment.

Stewart et al. (2000b) also compared the content of the highly chlorinated PCB homologs or isomers with 7, 8, or 9 chlorines (septa-, octa-, and nonyl-chlorinated biphenyls) in the mothers’ blood at the end of the first trimester with their fish diet. The mothers who never ate fish from the lakes had the lowest concentrations of the highly chlorinated PCBs in their blood. In 1984 when the first fish advisories were issued warning pregnant women not to eat the fish from Lake Ontario, the mothers who stopped eating fish had less of the isomers than the mothers who only stopped eating Lake Ontario fish when they found they were pregnant. The mothers who did not stop eating fish throughout their pregnancies had the highest PCB isomer concentrations (Stewart et al. 1999). Stewart et al. (2000a) found a dose–response relationship between the prenatal exposure of the children to the highly chlorinated PCBs and increases in their reflexive and autonomic deviations from the norm and their reduced ability to habituate under various conditions. MRI examination of the most highly exposed children in this study revealed an inverse dose–response association between their PCB cord blood and the volume/size of the splenium of the corpus callosum at 7.8 years of age, and their response inhibition at 4.5 years of age, a behavioral characteristic seen in ADHD children where they do not adapt well to their environment and have trouble settling down (Stewart et al. 2003). The splenium is the bridge between the right and left sides of the corpus callosum.

Another set of studies with healthy mothers and infants performed in the Netherlands examined a cross-section of the population, not just fish eaters (Koopman-Esseboom et al. 1996). This team found neuromuscular delays in the children at 3 months of age in association with *in utero* exposure to PCBs and dioxins measured as dioxin toxicity equivalents (TEQs) (Brouwer et al. 1995). Additionally, an inverse dose–response association was observed between increased TEQs with thyroid levels in the children and a positive association with unusual changes in their immune system (Weisglas-Kuperus et al. 1995). Further comparisons are difficult because the same battery of tests as those used in the United States was not employed in this series of studies.

The Netherlands research team divided the mothers into two groups, low TEQ (< 30.74 pg/g fat) and high TEQ (> 30.75 pg/g fat), on the basis of the equivalents in the plasma of the mother during the last month of pregnancy (Koopman-Esseboom et al. 1994). The differences between the two cohorts in total T_4_ (177.5–159.9 nmol/L) and thyroid-stimulating hormone (TSH) (1.9–2.6 μIU/mL) were significant at 2 weeks of age. However, the TSH levels of the mothers were within the normal range (3.0 IU/mL is the cutoff).

All the effects reported in the children in the studies described above were linked to the children’s prenatal experience. In each study mentioned, even though the decrements among the children were statistically significant at the population level, the parents or doctors of the infants would not have known they were compromised. It took skilled psychologists and technicians to quantify the changes in the children.

In the Lake Michigan study trained psychologists were able to measure developmental delays in infants shortly after birth if the blood fat of the mother held 1.00 parts per million (ppm) PCBs (Jacobson and Jacobson 1996). At 1.25 ppm PCBs, the change was statistically significant (*p* < 0.001) because there was so little variance. The intelligence and behavioral impairments reported in this study are populationwide. They are not rare events such as cancer. In this healthy mother–infant study, at 11 years of age, 11% of the children were affected (Jacobson and Jacobson 1996). At 4 years of age, 17 children were removed from the study because they were too hyperactive and would not take the tests (Jacobson et al. 1990). If the outliers had remained in the study, 20% of the children would have been affected. It was later determined that the children who were removed from the study were the children of the mothers with the highest PCB concentrations in the study. Another child was removed from the study at the end because he or she had an IQ below 70 (Jacobson and Jacobson 1996). These researchers noted that consuming fish is not the only source of PCBs, but these compounds are found in many other foods such as meats, fatty foods, fast foods, cheeses, ice cream, and even in the most rigid vegan diet (Schecter et al. 2001).

A Japanese group measured TEQs in breast milk at approximately 3 months postpartum and compared those with T_3_ and T_4_ in blood of the infants at 1 year of age (Nagayama et al. 1998). Only healthy mothers and full-term infants from southern Japan were selected for this study. There was a significant inverse relationship between total TEQs and T_3_ and T_4_ of the infants (*n* = 40). In this study, TSH had no association with the contaminants, suggesting that TSH may not be as sensitive an end point as previously considered without accompanying T_3_ and T_4_ measurements. No behavioral results accompanied these data. This study confirms, however, the transfer of contaminants, measured as TEQs, from the mother to the child and a change in the circulating thyroid hormones of the child distinguishable at the population level.

## Opening the Black Box of Exposure

The most difficult task for epidemiologists is to demonstrate exposure during development in human populations. Unlike the scenario where dioxin exposure was correlated with brain damage in great blue herons, in most epidemiologic studies attempting to determine etiology, the timing, scope of chemicals involved or range of exposure, and the actual body burdens of the chemicals are not knowable. Fortunately, in the past 5 years, technology for measuring human exposure to synthetic chemicals has advanced considerably. For example, the CDC can now monitor human blood and urine for > 116 chlorinated and nonchlorinated chemicals and their metabolites. These include contemporary-use pesticides and industrial chemicals used in cosmetics, perfumes, detergents, toys, plastics, and fire retardants—many of which are high-production-volume chemicals widely used in commerce (Burse et al. 1996; CDC 2003).

CDC chemists have begun to open the black box of exposure not only for a better picture of human organochlorine chemical exposure but for a number of other widely used chemicals that have not been studied as intensely. For example, they discovered that some metabolites of a class of chemicals called phthalates were 9 times higher in the urine of women between 20 and 40 years of age—women of childbearing age—than in any other segment of the population (Blount et al. 2000). Phthalates make plastics flexible and soft; they are used to improve delivery systems in perfumes, nail polish, shampoos, cosmetics, and dermal and intravenous applications of medications. They have been widely used as inert ingredients in pesticide formulations. Three of the phthalates, diethylhexyl phthalate, di(*n*-octyl) phthalate, and di(*n*-hexyl) phthalate, are antiandrogens in laboratory animals, producing hypospadias, cryptorchidism, and other male developmental disorders. They also interfere with the thyroid system (Hinton et al. 1977; Price et al. 1988).

## Discussion

Increases in the prevalence of neurodevelopmental disorders over the past 30 years make it imperative to reverse this trend. Because it appears that this trend could be partly the result of exposure to environmental contaminants, it is also imperative to prevent further exposure to synthetic chemicals that are suspect. Fortunately, technological improvements in the past 10 years have broadened the scope and sensitivity of detection for not only synthetic chemicals in human tissues but also for natural endogenous hormones. The evidence that certain hormones operate at parts per trillion and parts per billion and equivalent exposure to endocrine-active chemicals is equivalent or higher reveals the extreme vulnerability of development to chemical perturbation. This detection technology, combined with large-scale epidemiologic studies, is beginning to reveal a panorama of subtle biological differences within populations that would never be recognized at the individual level. The work of Haddow et al. (1999) is an example of what we can learn by stepping back and observing what is happening in the population. Their work suggests that *a*) it is time to reassess what is considered euthyroid; *b*) quantification of T_4_,fT_4_, T_3_, and fT_3_ is needed to determine the thyroid hormone status of a pregnant woman; and *c*) routine maternal thyroid hormone monitoring throughout gestation should become standard practice.

It is important to note that increasing numbers of children are exhibiting attention deficit disorder and ADHD-like symptoms in the classroom, in turn placing more and more responsibility on teachers, families, social services, and taxpayers. A new approach for exploring the etiology of these disorders is needed that could include the use of more comprehensive case histories for the entire family. For example, the road map of brain/thyroid development presented by Howdeshell (2002) provides a starting place for specifically seeking the etiology of neurodevelopmental disorders. A vertical dissection of Figure 1 at various times throughout gestation cuts through many brain development events occurring simultaneously that are thyroid dependent. If a chemical were to interfere with the commencement of development of the cochlea at 6 weeks through its antithyroid effect, it might also affect other thyroid-sensitive tissues emerging at the same time. This might partly explain the list of irregularities or sequelae of anomalies that fit within the definition of the ADHD and autism syndromes. Overlay maps must be constructed of other developing systems to determine where the presence of a foreign chemical in the womb could interfere simultaneously with the developing brain and thyroid system. Comorbidities should begin to appear on these overlays, for example, the relationship between autism and hypospadias at 7–8 weeks when sexual differentiation commences at the same time as the development of the urogenital system and the hippocampus. Could one or more of the chemicals that are known antiandrogens and antithyroidals be involved in the latter? These junctures could be the tip of the iceberg in terms of determining the etiology of a disorder. To take advantage of this approach, more extensive case histories of autistic children are needed to determine other anomalies they are experiencing. In addition, exposure histories are needed of the parents of children with obvious developmental disorders, with emphasis on their lifestyle and occupational exposure before the birth of their child, not just focusing on the early postnatal and immediate exposure of the child.

Because it is now the rule to demand only statistically significant results in studies, a great deal of insight can be lost, as was the case with the Lake Michigan studies. It is time to reassess how statistics are applied in health-related studies and the conclusions used in risk analyses. For example, because an average IQ deficit of 6 points is within two standard deviations of normal, it could be dismissed as an adverse effect. If this statistical criterion were applied, it would exclude the most sensitive but still responding individuals. However, when viewed from the population level, this can have a tremendous impact on the economy and integrity of a society. Unfortunately, the true robustness of the Lake Michigan PCB studies mentioned previously is lost in the final statistical analysis for several reasons. First, because only the healthiest pairs of infants and mothers were used in any of the these studies, the most vulnerable segment of the population was eliminated before the study started. Second, by eliminating the outliers (*n* = 18), another vulnerable segment of the population was removed. In so doing, the overall population effect was artificially reduced, thus minimizing the importance and significance of the study results. It is time for a thorough reassessment of statistical applications and study designs in long-term, large-scale epidemiologic studies to fully assess damage in the entire population, not only in representative subpopulations. This would assist those concerned about etiology and prevention.

During the organizational stages of gestation, responses to endocrine disruption are unlike the typical responses in adulthood. Consequently, testing with mature animals misses the organizational damage from prenatal exposure. In addition, most traditional toxicological tests use doses 1,000–1,000,000 times that of the equivalent physiological range at which the endocrine systems operate and well above real-world exposure concentrations to synthetic chemicals. The high doses used in toxicological testing far exceed the normal threshold or peak concentrations at which homeostatic negative-feedback control from the brain shuts down cellular responses. Consequently, other nonendocrine toxic effects might be expressed in exposed adult animals but not the same effects if exposure had taken place during their construction and programming. Thus, in endocrine disruption, extrapolating down from several high doses to determine the lowest safe dose or no-effect dose of a chemical will not protect the fetus. Fortunately, many innovative and entirely new protocols for detecting endocrine disruption are in the early stages of being validated and standardized in dozens of countries around the world, but unfortunately, it will take years before many will be ready for use.

## Figures and Tables

**Table 1:** Chronology of human exposure.

Years	Exposure scenario
1920s–1930s	BPA, PCBs, and DDT commercially introduced. Chlorine industry expanding. Discrete postnatal and prenatal exposure.
1940s–WWII	First wide-scale production and exposure to the above and other chemicals including plastics and chlorinated compounds as technology advanced.
1940s–1950s	First generation widely exposed postnatally and some who may have been exposed prenatally.
1950s–1970s	First generation born that was widely exposed prenatally.
1970s–1990s	First generation that was widely exposed prenatally reached reproductive age.
1980s–present	Second generation born that was exposed in the womb and beginning to produce the third generation. Production volume and exposure still increasing.
